# Use of Term *Excited Delirium* in State EMS Protocols Over Time

**DOI:** 10.1001/jamanetworkopen.2024.19183

**Published:** 2024-06-28

**Authors:** Christie L. Fritz, David W. Schoenfeld, Jake D. Hoyne, Stephen H. Thomas

**Affiliations:** 1 Department of Emergency Medicine, Beth Israel Deaconess Medical Center and Harvard Medical School, Boston, Massachusetts; 2 Blizard Institute, Barts and London School of Medicine, London, United Kingdom

## Abstract

This cross-sectional study investigates changes in use of the term *excited delirium* in state emergency medical services (EMS) protocols after professional society statements condemning the term.

## Introduction

*Excited delirium* (ExD) is a controversial and poorly defined term.^1,2^ After incidents involving law enforcement and emergency medical services (EMS), the American Psychiatry Association (APA) stated in 2020 that ExD terminology is “too nonspecific to meaningfully describe and convey information about a person” and “should not be used until a clear set of diagnostic criteria are validated.”^1^

ExD terminology includes characterizations of aggression, erratic behavior, and superhuman strength and is more commonly applied to minority racial and ethnic populations.^2,3^ Subsequent statements by the American Medical Association (AMA) and other organizations^3,4^ echoed APA concerns over this bias. After high-profile poor patient outcomes in 2023, media attention increased, California banned the term as a diagnosis, and additional societies condemned the terminology.^5^

This study assesses whether US EMS statewide treatment protocols (STPs) reflect condemnation of the term. We aimed to assess whether STPs ceased using the term after the AMA statement and in 2024, after increased media attention and condemnation.

## Methods

This cross-sectional study identified 32 publicly available STPs from internet searches in August 2023 and March 2024 (eMethods in Supplement 1). STPs could be mandatory or guidelines (eg, intended for reference or adaptation by EMS groups within the state). We categorized states by the presence of an STP, date of most recent update, and presence of the term *excited delirium*. We determined ExD use if the term appeared in the protocol title or discussion but not when referenced as historical or undesirable.

*Recent updates *were defined a priori as those occurring after December 31, 2021, the year after the AMA position statement. We reexamined STPs in March 2024 to evaluate if ExD had been removed after national attention.

The Fisher exact test assessed univariable associations between ExD term usage and STP updates. Stata version 18MP (StataCorp) was used for analysis.

## Results

Of 50 states and Washington, District of Columbia, STPs were identified for 32 states (62.7%), of which 21 STPs (65.6%) contained ExD terminology in 2021 to 2023 (Figure). An additional 7 STPs (21.8%) removed the terminology in 2023 to 2024 updates, leaving 43.8% of states still including ExD.

**Figure.**
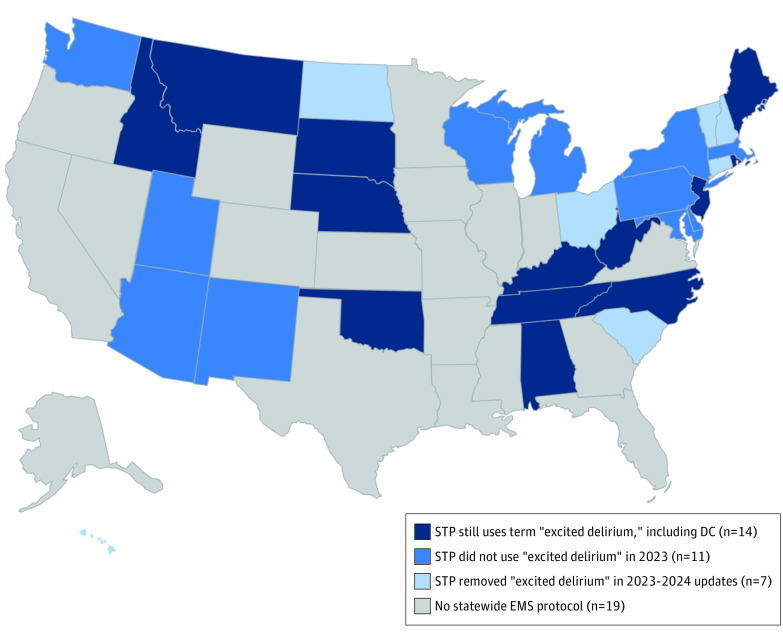
Excited Delirium Terminology in Statewide Emergency Medical Services (EMS) Protocols DC indicates District of Columbia; STP, statewide treatment protocol.

ExD terminology was significantly less likely to appear in STPs with updates after the 2020 AMA statement. ExD terminology appeared in 12 of 13 STPs last updated in 2021 or earlier (92.3%) and 9 of 18 STPs updated after 2021 (50.0%) (*P* = .02). In 2024, 9 of 12 STPs most recently revised before 2023 (75.0%) and 5 of 20 protocols most recently revised in 2023 to 2024 (25.0%) used the term. Recent protocol revision (2023 or 2024) was associated with lower likelihood of using the term (*P* = .01).

## Discussion

ExD has been characterized as lacking medical accuracy and being historically subject to misapplication.^1,3,6^ It has been used to justify inappropriate restraint based on race and ethnicity, gender, and mental illness.^3,6^ Despite being inconsistent with patient-centered prehospital care and evidence, ExD terminology appeared in the almost half of US STPs. There was a large decrease in ExD use in STPs after media attention increased, California banned the term, and the American College of Emergency Physicians released an April 2023 statement condemning the term.^5^

Study limitations include use of publicly available STPs, which may not have captured all STP updates removing ExD, and that no STP was identified for 19 US states, which may have local or regional protocols including ExD. However, persistence in some STPs suggests that the term has not been fully extinguished from the prehospital lexicon.

Despite multiple position statements by national organizations, use of the term persisted, although its use decreased, especially after 2023. States may consider alternative terminology (eg, hyperactive delirium)^5^ or further clarification around medical, toxicologic, or behavioral causes for this overly broad patient presentation. Attention is warranted to encourage further alignment between STPs and professional society recommendations to reject ExD terminology.

## Notes

Supplement 1. **eMethods.** Research Methods Expanded

Supplement 2.
Data Sharing Statement

## References

[1] Council on Psychiatry and Law. Position statement on concerns about the use of the term “excited delirium” and appropriate medical management in out-of-hospital contexts. American Psychiatric Association. Accessed September 2, 2023. https://www.psychiatry.org/getattachment/7769e617-ee6a-4a89-829f-4fc71d831ce0/Position-Use-of-Term-Excited-Delirium.pdf

[2] Vilke GM, DeBard ML, Chan TC, . Excited delirium syndrome (ExDS): defining based on a review of the literature. J Emerg Med. 2012;43(5):897-905. doi:10.1016/j.jemermed.2011.02.017 21440403

[3] American Medical Association. New AMA policy opposes “excited delirium” diagnosis. Accessed September 2, 2023. https://www.ama-assn.org/press-center/press-releases/new-ama-policy-opposes-excited-delirium-diagnosis

[4] American College of Emergency Physicians Hyperactive Delirium Task Force. ACEP reaffirms positions on hyperactive delirium. Accessed May 22, 2024. https://www.acep.org/news/acep-newsroom-articles/aceps-position-on-hyperactive-delirium

[5] American College of Emergency Physicians Hyperactive Delirium Task Force. ACEP reaffirms positions on hyperactive delirium. Accessed March 31, 2024. https://www.acep.org/siteassets/new-pdfs/education/acep-task-force-report-on-hyperactive-delirium-final.pdf

[6] McGuinness T, Lipsedge M. ‘Excited delirium’, acute behavioural disturbance, death and diagnosis. Psychol Med. 2022;52(9):1601-1611. doi:10.1017/S0033291722001076 35546291 PMC9280280

