# Supplementary material for: Use of Term Excited Delirium in State EMS Protocols Over Time
Source: JAMA Netw Open. 2024 Jun 28;7(6):e2419183. doi: 10.1001/jamanetworkopen.2024.19183 (PMC11214108; doi:10.1001/jamanetworkopen.2024.19183)
Supplement: Supplement 1. — eMethods. Research Methods Expanded [file jamanetwopen-e2419183-s001.pdf]

Supplemental Online Content

Fritz CL, Schoenfeld DW, Hoyne JD, Thomas SH. Use of term *excited delirium* in state EMS protocols over time. *JAMA Netw Open*. 2024;7(6):e2419183. doi:10.1001/jamanetworkopen.2024.19183

eMethods. Research Methods Expanded

This supplemental material has been provided by the authors to give readers additional information about their work.

© 2024 Fritz CL et al. *JAMA Network Open.*

eMethods. Research Methods Expanded

Publicly available Statewide Treatment Protocols (STPs) were identified from an internet search (August 2023) by search engines Google and Bing and evaluating individual state websites. This was performed again in March 2024 to evaluate change over time. STPs could either be mandatory or guidelines (*e.g.* intended for reference or adaptation by EMS groups within the state), with local or regional guidelines excluded. States that have the National Association of State EMS Officials (NASEMSO) National Model EMS Clinical Guidelines as their only guideline for protocols (N=4) were not included as a statewide EMS protocol. Scope of practice documents were also not included. We also excluded states with solely resource documents, single protocols (ie stroke or trauma) only or non-structured protocols/scope of practice.

We categorized each state by the presence of an STP, if it was recently updated, and presence of the term “excited delirium”. Only the term “excited delirium” was utilized as it is the exact term that has been seen as problematically used and specifically condemned by national medical organizations. Other terms/diagnoses have not similarly been identified on a national scale in the same way that “Excited delirium” has. Terms such as “agitated delirium”, “hyperactive delirium” or “delirium with agitated behavior” were not defined as a positive in our analysis as they were seen as attempts to move away from “excited delirium” as a specific term.

© 2024 Fritz CL et al. *JAMA Network Open.*
