# Supplementary material for: Use of Term Excited Delirium in State EMS Protocols Over Time
Source: JAMA Netw Open. 2024 Jun 28;7(6):e2419183. doi: 10.1001/jamanetworkopen.2024.19183 (PMC11214108; doi:10.1001/jamanetworkopen.2024.19183)
Supplement: Supplement 2. — Data Sharing Statement [file jamanetwopen-e2419183-s002.pdf]

Data Sharing Statement

Fritz. Use of Term Excited Delirium in State EMS Protocols Over Time. *JAMA Netw Open*. Published June 28, 2024. doi:10.1001/jamanetworkopen.2024.19183

Data
Data available: Yes
Data types: Data (not involving human participants)
How to access data: Email cfritz@bidmc.harvard.edu for any data questions/requests
When available: With publication

Supporting Documents
Document types: Other (please specify)
Additional Information: Any requested data or statistical information is available
How to access documents: Email cfritz@bidmc.harvard.edu for any data questions/requests
When available: With publication

Additional Information
Who can access the data: Any time
Types of analyses: For Any purpose
Mechanisms of data availability: Email cfritz@bidmc.harvard.edu for any data questions/requests
Any additional restrictions: None
